# Higher dietary vitamin D intake influences brain and mental function in elderly Americans: a cross-sectional analysis

**DOI:** 10.3389/fnut.2025.1564568

**Published:** 2025-04-09

**Authors:** Huizhen Chen, Xing Pang, Yinhui Huang

**Affiliations:** ^1^Department of Laboratory, Jinjiang Municipal Hospital, Quanzhou, China; ^2^Department of Neurology, Jinjiang Municipal Hospital, Quanzhou, China

**Keywords:** vitamin D, cognitive function, depression, elderly, NHANES

## Abstract

**Background:**

Vitamin D is known to have a potential impact on cognitive function and mental health. This study aims to assess the association between dietary vitamin D intake and cognitive performance, as well as depression, in an elderly U.S. population.

**Methods:**

Data from the National Health and Nutrition Examination Survey (NHANES) 2013–2014 were analyzed. A total of 1,344 elderly participants were categorized into three tertiles based on their dietary vitamin D intake (D2 + D3). Cognitive function was measured using the Consortium to Establish a Registry for Alzheimer’s Disease (CERAD) test, Digit Symbol Substitution Test (DSST), and Animal Fluency Test, while depression was assessed through the Patient Health Questionnaire-9 (PHQ-9). Adjustments were made for confounding variables, including age, sex, race, education, physical activity level, and other dietary factors.

**Results:**

After adjustment for confounders, individuals in the 3rd tertile of vitamin D intake (≥4.9 mcg/day) had significantly reduced odds of low performance on the CERAD test (OR: 0.77, 95% CI: 0.57–0.98; *p* = 0.031) and Animal Fluency test (OR: 0.63, 95% CI: 0.49–0.85; *p* = 0.013) compared to the 1st tertile of intake (≤2.4 mcg/day). Similarly, participants in the 3rd tertile of vitamin D intake had lower odds of depression (PHQ-9 score > 4) after adjustment (OR: 0.68, 95% CI: 0.48–0.99; *p* = 0.046).

**Conclusion:**

Our findings suggest that dietary vitamin D intake is associated with improved cognitive function and depressive symptoms in elderly individuals. However, further longitudinal studies are needed to establish causality and explore the underlying mechanisms.

## Introduction

The global prevalence of dementia is rapidly increasing, with an estimated 55 million people affected in 2020, a number projected to triple by 2050 due to population aging (1). Similarly, depression, which affects approximately 10–20% of older adults globally, is expected to rise as life expectancy increases and mental health challenges become more prevalent among the elderly (2). These alarming trends emphasize the urgent need for identifying modifiable risk factors and developing cost-effective interventions such as dietary interventions to diminish the burden of these debilitating conditions.

Vitamin D, a fat-soluble vitamin primarily obtained from limited dietary sources and sunlight exposure, plays a pivotal role in numerous physiological processes, including calcium homeostasis, bone health, and immune modulation (3). Beyond its traditional functions, emerging evidence has highlighted its potential role in cognitive and mental health (4). Decline of cognitive function and depression are significant public health concerns, particularly among older adults (5), who are most vulnerable to deficiencies in vitamin D due to reduced skin synthesis, dietary insufficiencies, and lifestyle changes associated with aging (6).

The brain expresses vitamin D receptors (VDR) and enzymes involved in its metabolism (7), suggesting its active role in neural function. Vitamin D has been implicated in neuroprotection, modulation of neuroinflammation, regulation of neurotrophic factors, and maintenance of neurotransmitter homeostasis (8, 9). While these associations are biologically plausible, findings from observational and interventional studies remain inconsistent, warranting further exploration (10–13). Moreover, cognitive function and depression share a bidirectional relationship, where impairment in one domain can exacerbate issues in the other, creating a complex and intertwined dynamic. Cognitive decline may lead to feelings of hopelessness and depression due to a loss of independence and functionality, while depression can adversely affect cognitive performance through mechanisms such as increased inflammation, increased cortisol levels, altered neurotransmitter activity, and impaired neurogenesis (14, 15). A cohort of 8,268 participants found that higher depressive symptoms were associated with poorer memory and verbal fluency. Furthermore, a linear decline in memory was linked to an acceleration of depressive symptoms over time (16). Other studies have supported these findings (17–20). Despite the recognition of this intricate interplay, the precise pathways connecting these conditions remain incompletely understood.

A growing body of research has explored the roles of vitamin D in cognitive function and depression among older adults. A systematic review and meta-analysis of observational studies found that lower vitamin D levels were associated with a higher risk of depression in adults (21). However, a study in Netherland found no significant association between vitamin D levels and the course of depression or remission rates (22). A cross-sectional analysis on American elderly found that vitamin D intake was positively associated with cognitive performance (23). However, a systematic review concluded that vitamin D supplementation did not provide clear cognitive benefits, with findings varying across different studies (24). Despite numerous studies examining the effects of vitamin D on depression and cognitive function separately, comprehensive analyses focusing on its association with both outcomes simultaneously in older adults remain limited. Performing a study that address both outcomes could strengthen the understanding of dual role of vitamin D in mental health among older adults.

This study aims to address this gap by examining the relationship between dietary vitamin D intake and cognitive and mental function among elderly Americans using the National Health and Nutrition Examination Survey (NHANES) 2013–2014 data. Cognitive function is assessed using standardized tests, including the Consortium to Establish a Registry for Alzheimer’s Disease (CERAD) test, the Animal Fluency test, and the Digit Symbol Substitution Test (DSST), while mental health outcomes are evaluated through the Patient Health Questionnaire-9 (PHQ-9). By shedding light on whether vitamin D influences cognitive or mental health or both, this study aims to unravel this intricate association and clarify role of vitamin D in these domains. The findings from this research could inform public health policies, dietary recommendations, and future interventional studies aimed at promoting cognitive resilience and mental well-being in older adults.

## Materials and methods

### Study design and population

This cross-sectional study utilizes data from the National Health and Nutrition Examination Survey (NHANES) 2013–2014, a nationally representative survey conducted by the Centers for Disease Control and Prevention (CDC).^1^ For this analysis, we included participants aged 60 years and older who completed dietary assessments, cognitive function tests, and mental health evaluations. Participants with missing data on key variables were excluded. A total of 1,344 survey participants who had sufficient information on vitamin D intake, cognitive function, and mental health were included in our analysis. The NHANES protocol was reviewed and approved by the National Center for Health Statistics (NCHS) Research Ethics Review Board. Written informed consent was obtained from all participants.

### Dietary vitamin D intake assessment

Dietary vitamin D intake was assessed using two 24-h dietary recall interviews. The first recall was conducted in person, and the second was collected via telephone. Dietary intake data were processed using the United States Department of Agriculture (USDA) Food and Nutrient Database for Dietary Studies (FNDDS) to calculate total daily vitamin D intake. Supplement use was not included in this analysis due to varying composition, dosage, and bioavailability of supplements, making it difficult to standardize their effects. Details of NHANES dietary analyses are reported in the following link: https://wwwn.cdc.gov/nchs/nhanes/tutorials/dietaryanalyses.aspx.

### Cognitive function assessment

Cognitive function was evaluated using three standardized tests: Consortium to Establish a Registry for Alzheimer’s Disease (CERAD) Word Learning Test, Digit Symbol Substitution Test (DSST), and Animal Fluency Test. CERAD assesses immediate and delayed memory by asking participants to recall a list of 10 words over three trials and again after a delay (typically during which other tasks are completed). The total score of immediate recall is the sum of correctly recalled words across all three trials, ranging from 0 to 30. The delayed recall score ranges from 0 to 10 based on the number of words correctly recalled. The Animal Fluency Test measures verbal fluency and executive function by asking participants to name as many animals as possible within 60 s. The score is the total number of unique animals correctly named. DSST evaluates processing speed, attention, and working memory by requiring participants to match symbols to numbers according to a provided key within 120 s. The score is the total number of correct matches completed within the time limit, ranging from 0 to a maximum score (typically around 133, depending on the test version). In all tests, higher scores indicate better cognitive function (25). Scores from these tests were analyzed individually and as a composite measure of overall cognitive function. Based on earlier published research, we established the lowest tertile of the scores as the cut-off value (26, 27).

### Mental health assessment

Mental health status was assessed using the Patient Health Questionnaire-9 (PHQ-9), a validated tool for screening depression severity. The PHQ-9 includes nine items that evaluate the frequency of depressive symptoms over the past 2 weeks, with scores ranging from 0 (no symptoms) to 27 (severe depression). Scores 0–4 show minimal or no depression and scores>4 are related to depressive disorder, ranging from mild depression to severe depression (28).

### Covariates

Following assessing previous related researches, demographic, socioeconomic, and lifestyle factors were considered as covariates to account for potential confounding (23, 28). These variables included age, sex, race/ethnicity, educational level, marital status, income, body mass index, serum 25OHD2 + 25OHD3, energy intake, protein intake, carbohydrate intake and fat intake. The total metabolic equivalent (MET) score was determined by summing the MET values from five activity categories: vigorous recreational activities (MET = 8), vigorous occupational activities (MET = 8), moderate recreational activities (MET = 4), moderate occupational activities (MET = 4), and walking or bicycling for transportation (MET = 4). The MET score for physical activity was calculated as follows: Physical activity (MET score) = sum of [days per week of each activity × duration of each activity (minutes) × corresponding MET value]. The final MET scores were then grouped into four levels of activity: inactive (MET minutes/week <250), somewhat active (250 ≤ MET minutes/week <500), active (500 ≤ MET minutes/week <1,000), and very active (MET minutes/week ≥1,000) (29).

### Statistical analysis

Data were analyzed using the NHANES 2013–2014 dataset, accounting for the complex sampling design by applying appropriate survey weights, strata, and primary sampling units to ensure nationally representative estimates. Continuous variables were described as means and standard deviations, while categorical variables were presented as frequencies and percentages. Differences across dietary vitamin D intake tertiles were assessed using one-way ANOVA for continuous variables and chi-square tests for categorical variables. Significant differences between tertiles were identified with *post hoc* LSD tests.

To examine the association between dietary vitamin D (D2 + D3) intake and cognitive performance, weighted logistic regression models were used to calculate odds ratios (ORs) and 95% confidence intervals (CIs) for low cognitive performance on the CERAD, Animal Fluency, and DSST tests. Similarly, the relationship between dietary vitamin D intake and depressive symptoms (PHQ-9 items) was analyzed using weighted logistic regression. The crude model and adjusted models controlling for potential confounders (age, sex, race, educational level, physical activity level, marital status, income, BMI, serum 25OHD2 + 25OHD3 levels, and macronutrient intake) were fitted.

All analyses were conducted using SPSS software (V22; SPSS Inc., Chicago, IL), and *p*-values <0.05 were considered statistically significant. Statistically significant associations were highlighted in bold within the tables.

## Results

### Participant characteristics by tertiles of vitamin D intake

A total of 1,344 participants in the NHANES 2013–2014 study were categorized into tertiles based on their dietary vitamin D intake (D2 + D3). The distribution of gender, age, BMI, and dietary intake significantly varied across tertiles. The proportion of males increased with higher tertiles (40% in the 1st tertile to 56.1% in the 3rd tertile, *p* < 0.001). Participants in the highest tertile were older (70.28 ± 6.99 years) compared to those in the lowest tertile (68.87 ± 6.52 years, *p* = 0.006). BMI was significantly lower in the 3rd tertile compared to the 1st (28.56 ± 6 vs. 29.39 ± 6.35 kg/m^2^, *p* = 0.016). Energy, protein, carbohydrate, and fat intakes were markedly higher in the 3rd tertile compared to the 1st tertile (all *p* < 0.001). Serum vitamin D levels, however, showed no significant difference between tertiles (*p* = 0.11) (Table 1).

**Table 1:** Characteristics of participants in different quartiles of VD (D2 + D3) intake, NHANES 2013–2014.

	Vitamin D (D2 + D3) intake tertiles			
	1st (<2.4 mcg)	2st (2.4–4.9 mcg)	3st (4.9 > mcg)	*p*-value
Gender				
Male	178 (40)	209 (46.4)	252 (56.1)	<0.001
Female	267 (60)	241 (53.6)	197 (43.9)	
Age (years)	68.87 (6.52)	69.88 (6.67)^a^	70.28 (6.99)^a^	0.006
Weight (kg)	79.37 (19.17)	81.37 (20.24)	79.63 (19.56)	0.25
BMI (kg/m^2^)	29.39 (6.35)	29.76 (6.58)	28.56 (6)^ab^	0.016
Serum 25OHD2 + 25OHD3 (nmol/L)	75.15 (34.81)	77.34 (32.12)	79.67 (29.11)	0.11
Energy intake (kcal/day)	1549.3 (601.3)	1770.6 (611.8)^a^	2131.6 (688.1)^ab^	<0.001
Protein (g/day)	59.71 (25.14)	70.22 (25.35)^a^	88.35 (30.88)^ab^	<0.001
Carbohydrate (g/day)	186.16 (76.24)	214.87 (77.1)^a^	256.91 (86.94)^ab^	<0.001
Fat (g/day)	60.37 (30.82)	69.05 (31.49)^a^	82.19 (34.41)^ab^	<0.001
Race				
Hispanic	80 (18)	94 (20.9)	79 (17.6)	0.003
Non-Hispanic White	213 (47.9)	241 (53.6)	263 (58.6)	
Non- Hispanic Black	110 (24.7)	89 (19.8)	70 (15.6)	
Other Races	42 (9.4)	26 (5.8)	37 (8.2)	
Education level				
< high school	103 (23.1)	106 (23.6)	88 (19.6)	0.54
High school	111 (24.9)	111 (24.7)	109 (24.3)	
College graduate ≤	231 (51.9)	232 (51.7)	252 (56.1)	
Marital status				
Married	266 (59.8)	265 (59)	271 (60.4)	0.95
Widowed/Divorced/Separated	157 (35.3)	158 (35.2)	157 (35)	
Never married	22 (4.9)	26 (5.8)	21 (4.7)	
Income (%FIPR)				
<130%	112 (27)	129 (30.6)	114 (27)	0.39
130–349%	175 (42.2)	160 (38)	160 (37.8)	
≥350%	128 (30.8)	132 (31.4)	149 (35.2)	
Physical activity status				
Inactive	156 (35.1)	179 (39.8)	180 (40.1)	0.12
Somewhat active	74 (16.6)	94 (20.9)	72 (16)	
Active	93 (20.9)	70 (15.6)	81 (18)	
Very active	122 (27.4)	107 (23.8)	116 (25.8)	

Values were described as mean (standard deviation) for continuous variables and frequency (%) for categorical variables. NHANES, National Health and Nutrition Examination Survey; BMI, body mass index; FIPR, family income to poverty level ratio. *p*-values were calculated by using the one-way ANOVA test for continuous variables and chi-squared for categorical variables. ^a^ *p* < 0.05 compared to tertile 1, ^b^ *p* < 0.05 compared to tertile 2.

### Association between vitamin D intake and brain function

After adjustment for confounders, individuals in the 3rd tertile of vitamin D intake had significantly reduced odds of low performance on the CERAD test (OR: 0.77, 95% CI: 0.57–0.98; *p* = 0.031) and Animal Fluency test (OR: 0.63, 95% CI: 0.49–0.85; *p* = 0.013) compared to the 1st tertile of vitamin D intake. No significant association was observed between vitamin D intake and low performance on the DSST test in the crude and adjusted models (Table 2).

**Table 2:** Weighted ORs (95%CI) for association of low cognitive performance (CERAD test, Animal Fluency test and DSST) across dietary VD intake, NHANES 2013–2014 (*N* = 1,344).

	Vitamin D (D2 + D3) Intake Tertiles		
	1st (<2.4 mcg)	2st (2.4–4.9 mcg)	3st (4.9 > mcg)
CERAD test (Low performance)			
Crude	1	1.2 (0.98–1.47)	0.87 (0.71–1.07)
Adjusted*	1	1.07 (0.83–1.34)	**0.77 (0.57–0.98)**
Digit symbol test (Low performance)			
Crude	1	1.25 (0.99–1.58)	0.99 (0.78–1.58)
Adjusted*	1	1.19 (0.91–1.54)	1.23 (0.92–1.67)
Animal fluency test (Low performance)			
Crude	1	1.19 (0.97–1.47)	**0.66 (0.53–0.82)**
Adjusted*	1	1.13 (0.88–1.46)	**0.63 (0.49–0.85)**

Values represent adjusted odds ratios with 95%CI. Values in bold are statistically significant (*p* < 0.05). NHANES, National Health and Nutrition Examination Survey; CERAD, Consortium to Establish a Registry for Alzheimer’s disease. *Adjusted for age, sex, race, educational and physical activity level, marital status, income, body mass index, serum 25OHD2 + 25OHD3, energy intake, protein intake, carbohydrate intake and fat intake.

### Association between vitamin D intake and depression symptoms

Dietary vitamin D intake showed an inverse association with specific depression symptoms and overall depressive status. Individuals in the 3rd (OR: 0.45, 95% CI: 0.26–0.76; *p* = 0.006) and 2st tertiles (OR: 0.58, 95% CI: 0.36–0.89; *p* = 0.011) had significantly reduced odds of experiencing thoughts of being better off dead compared to the 1st tertile, even after adjustment. Additionally, participants in the 3rd tertile had lower odds of depression (PHQ-9 score > 4), even after adjustment (OR: 0.68, 95% CI: 0.48–0.99; *p* = 0.046). However, there was a significant positive association between higher dietary vitamin D intake (3rd title) and the likelihood of trouble sleeping or sleeping too much (OR: 1.36, 95% CI: 1.11–1.66; *p* = 0.033), following adjustment for confounding factors. Other depression-related symptoms, such as, appetite disturbances, energy levels, and feelings of worthlessness, did not show significant associations after adjustment (Table 3).

**Table 3:** Weighted ORs (95%CI) for association of depression (PHQ9 items) across dietary VD intake, NHANES 2013–2014 (*N* = 1,344).

	Vitamin D (D2 + D3) intake tertiles		
	1st (<2.4 mcg)	2st (2.4–4.9 mcg)	3st (4.9 > mcg)
Have little interest in doing things			
Crude	1	0.99 (0.82–1.2)	0.91 (0.75–1.1)
Adjusted*	1	1.1 (0.89–1.36)	1.18 (0.93–1.48)
Feeling down, depressed, or hopeless			
Crude	1	0.85 (0.69–1.03)	0.78 (−0.64–0.95)
Adjusted*	1	0.94 (0.75–1.16)	0.99 (0.78–1.24)
Trouble sleeping or sleeping too much			
Crude	1	0.96 (0.81–1.16)	1.16 (0.98–1.38)
Adjusted*	1	1.1 (0.93–1.31)	**1.36 (1.11–1.66)**
Feeling tired or having little energy			
Crude	1	1.02 (0.86–1.21)	0.97 (0.82–1.15)
Adjusted*	1	1.05 (0.86–1.26)	1.07 (0.85–1.29)
Poor appetite or overeating			
Crude	1	1.14 (0.93–1.4)	0.81 (0.66–1)
Adjusted*	1	1.19 (0.98–1.47)	0.95 (0.74–1.21)
Feeling bad about yourself			
Crude	1	1.13 (0.9–1.43)	1.09 (0.87–1.38)
Adjusted*	1	1.14 (0.91–1.48)	1.12 (0.86–1.43)
Trouble concentrating on things			
Crude	1	1.04 (0.83–1.3)	1.17 (0.94–1.45)
Adjusted*	1	1.06 (0.85–1.31)	1.21 (0.98–1.51)
Moving or speaking slowly or too fast			
Crude	1	0.83 (0.63–1.1)	0.98 (0.75–1.27)
Adjusted*	1	0.86 (0.64–1.13)	0.94 (0.68–1.29)
Thought you would be better off dead			
Crude	1	**0.58 (0.36–0.89)**	**0.44 (0.28–0.69)**
Adjusted*	1	**0.56 (0.33–0.91)**	**0.45 (0.26–0.76)**
Depression (PHQ-9 score > 4)			
Crude	1	1.05 (0.79–1.4)	**0.48 (0.49–0.92)**
Adjusted*	1	1.14 (0.83–1.58)	**0.68 (0.48–0.99)**

Values represent adjusted odds ratios with 95%CI. Values in bold are statistically significant (*p* < 0.05). Abbreviations: NHANES, National Health and Nutrition Examination Survey; PHQ9, Patient Health Questionnaire-9. *Adjusted for age, sex, race, educational level and physical activity level, marital status, income, body mass index, serum 25OHD2 + 25OHD3, energy intake, protein intake, carbohydrate intake and fat intake.

## Discussion

Our study investigates the association between dietary vitamin D intake and cognitive and mental function among elderly Americans, utilizing data from the NHANES 2013–2014 cycle. Our results indicated that dietary intake of vitamin D, independent of vitamin D serum level, is related to brain function and mental health. However, it is important to note that, due to the observational design of our study, the associations observed between dietary vitamin D intake and cognitive function are correlative rather than causal. Moreover, it is important to acknowledge that serum vitamin D levels do not always directly reflect dietary intake, as they are influenced by multiple factors, including sun exposure, supplementation, variations in hepatic and renal conversion of vitamin D to its active forms, and genetic variations such as polymorphisms in genes related to vitamin D transport (e.g., GC encoding vitamin D binding protein) and metabolism (e.g., CYP2R1, CYP27B1) (30–33). While our findings suggest a potential role of dietary vitamin D in cognitive and mental health, this association may be mediated through alternative mechanisms beyond serum levels. Vitamin D-rich foods, such as fatty fish, fortified dairy products, and egg yolks, are integral components of dietary patterns that support cognitive health (34). For example, the Mediterranean diet, abundant in vitamin D sources, has been associated with a lower risk of cognitive decline and depression (35). This association may result from synergistic interactions among nutrients. A clinical trial study suggested that combined intake of these vitamin D and omega-3 may offer superior protection against depression, anxiety, and sleep compared to individual nutrients alone (36). Moreover, emerging research indicates that vitamin D influences the gut microbiota, which in turn affects brain health (37, 38). Vitamin D receptors are present in the gut, and vitamin D deficiency has been linked to dysbiosis, an imbalance in gut microbial communities (39). Such imbalances have been associated with mood disorders and cognitive decline (40). Therefore, vitamin D intake may modulate gut microbiota composition, thereby impacting mental health and cognitive function, independent of vitamin D serum level, warranting further investigation into these pathways.

BMI, age, gender, intakes of macronutrients, and race were significantly different among tertiles of vitamin D intake. The possible effect of these confounders on the final results was minimized by adjusting in the final regression model. BMI is a well-known factor that can influence both cognitive function and mental health outcomes, with lower BMI often associated with improved cognitive performance and a reduced risk of depression (41). However, extreme BMI values, whether low or high, may also signal underlying health issues, such as frailty or metabolic disturbances, that could independently affect cognitive and mental health outcomes (41). By adjusting for BMI in our models, we aimed to isolate the effects of vitamin D intake on cognitive and mental health outcomes. However, differences in BMI could still carry important biological implications. Therefore, future studies should explore these associations further by investigating how changes in BMI over time or extreme values may directly influence cognitive and mental health outcomes, independent of other dietary as well as lifestyle factors.

The cognitive domains influenced by dietary vitamin D intake in this study included verbal fluency and episodic memory, as assessed by the Animal Fluency test and CERAD test, respectively. Individuals in the highest tertile of vitamin D intake showed significantly better performance in the Animal Fluency test, indicating enhanced verbal fluency, which reflects executive function and lexical access. Similarly, a reduced likelihood of poor performance in the CERAD test among those with higher vitamin D intake suggests improved episodic memory, a domain crucial for recalling specific events or experiences. However, in the domain of processing speed and attention, as measured by DSST, no significant association was observed between vitamin D intake and test performance after adjusting for confounders. This highlights that while vitamin D intake may positively influence certain cognitive domains, its effects may not extend universally across all aspects of cognition. Further studies are needed to clarify these domain-specific effects and underlying mechanisms. In terms of depression, the protective effect of vitamin D intake appears specific to more severe depressive symptoms, such as suicidal ideation, while its association with milder symptoms or other domains of depression was not significant. However, we found that higher tertile of vitamin D intake was associated with trouble sleeping. A clinical trial study found that vitamin D status was negatively associated with melatonin secretion (42). On the other hand, various studies suggested beneficial effect of vitamin D in sleep disorders (43, 44). Our study method cannot show causal relationships. Therefore, additional longitudinal studies are needed to investigate the relationship between vitamin D and sleep disorders.

Our findings align with prior studies linking vitamin D status to cognitive and mental health. For example, Shu et al. highlighted the association between higher serum vitamin D levels and improved cognitive function in older American adults, with depressive symptoms mediating this effect in populations with diabetes (28). Similarly, another study observed a positive relationship between dietary vitamin D intake and cognitive function in elderly American populations, suggesting that sufficient vitamin D consumption could mitigate age-related cognitive decline. However, this study focused exclusively on cognitive function without exploring depression as a potential mediator (23). Controversially, a longitudinal study in Swedish men with an 18-year follow-up reported no significant relationship between plasma 25-hydroxyvitamin D [25(OH)D] concentrations, dietary vitamin D intake, or genetic predisposition for vitamin D synthesis and the risk of all-cause dementia, Alzheimer’s disease, vascular dementia, or cognitive decline (13). The discrepancy between our findings and those of Olsson et al. (13) may be attributed to several important differences in study design, population characteristics, and assessment methods. Firstly, Olsson et al. (13) used a 7-day dietary record and assessed cognitive function using the Mini-Mental State Examination (MMSE), whereas we used different dietary assessment tools and employed more comprehensive cognitive function tests. Additionally, the study by Olsson et al. (13) was conducted on a cohort of Swedish older men, whereas the NHANES cohort represents a more ethnically and racially diverse population. This diversity may lead to differing dietary patterns, vitamin D sources, and other cultural factors influencing cognitive function. Moreover, the adjustment models used in both studies differ. Olsson et al. (13) did not adjust for the effect of vitamin D serum levels or income, which could influence cognitive outcomes. In addition, Olsson et al. (13) reported Hazard Ratios (HR) to estimate the risk of dementia, which accounts for time-to-event data, while our study used OR. Unlike HR, which measures the risk of an event occurring over time, OR provides a measure of association between exposure and outcome at a specific point in time, which may lead to different interpretations of the relationship between vitamin D intake and cognitive outcomes. Other studies identified that serum vitamin D level was associated with cognitive function in a Dutch, Brazilian, and American elderly cohort (11, 12, 45). Unlike studies focused on serum levels, our work specifically assesses the practical impact of food-based vitamin D intake on cognitive and mental health outcomes. The serum vitamin D level is more dependent on sun exposure than dietary intake (46). Therefore, our finding regarding the association between dietary vitamin D intake and brain and mental health in American elderly population, independent of sun exposure, may highlight the potential for dietary strategies to promote cognitive and mental health.

Given the observational nature of our study, the proposed mechanisms through which dietary vitamin D intake may influence cognitive and mental health remain speculative. Dietary vitamin D intake can influence cognitive and mental health through various molecular mechanisms. Vitamin D activates receptors in the brain, such as vitamin D receptors (VDRs) (7), which regulate gene expression related to neurogenesis, synaptic plasticity, and neuronal survival (7, 47). It promotes the production of neurotrophic factors like brain derived neurotrophic factors (BDNF) and neural growth factor (NGF), essential for cognitive function and neuroprotection (48). Additionally, vitamin D helps maintain calcium homeostasis for synaptic transmission and plasticity (49), modulates neurotransmitter systems like serotonin and dopamine (50), and reduces oxidative stress and neuroinflammation by inhibiting nuclear factor-kappa-B (NF-κB) activation (51). It also supports the integrity of the blood–brain barrier (52) and influences epigenetic mechanisms, such as DNA methylation and histone modifications, which affect genes involved in brain health (53).

The strengths of our study include its use of a nationally representative dataset, rigorous adjustment for confounders, and dual focus on cognitive and mental health outcomes. However, certain limitations should be acknowledged. First, utilizing data from a decade ago presents certain limitations. While more recent NHANES data cycles are available, the 2013–2014 dataset was selected due to its specific inclusion of variables pertinent to our research focus on dietary vitamin D intake and cognitive function. Moreover, a recent study investigated the trend of vitamin D intake using NHANES survey cycles 2001–2018 and found that despite an increasing trend in dietary vitamin D intake from 2007–2008 to 2017–2018 among the elderly population, dietary vitamin D intake has decreased from 4.83 in 2013–2014 mcg to 4.67 mcg in 2017–2018. However, this decrease was not statistically significant (54). As a result, it is difficult to definitively comment on how the current pattern of vitamin D intake in the elderly American population differs from that in 2013–2014, and future studies should address this issue. Second, the cross-sectional design precludes causal inference, necessitating longitudinal studies to confirm our findings. While we observed significant associations between dietary vitamin D intake and cognitive outcomes, we cannot infer that vitamin D intake directly causes changes in cognitive function or depression. The observed associations may reflect reverse causality, where cognitive decline or depression could lead to altered dietary patterns, including reduced vitamin D intake, rather than vice versa. Third, dietary vitamin D intake was assessed using a two-day 24-h dietary recall, which may not fully capture long-term intake patterns. Although this method is commonly used in large-scale studies like NHANES, it is subject to recall bias and day-to-day variations in dietary intake. Fourth, in this study low cognitive performance defined as the lowest tertile of test scores based on previous research (26, 27). While this approach is common, variations in cutoff points may affect the classification of cognitive impairment and, in turn, the interpretation of findings. Fifth, the study is geographically restricted to the elderly population in the U.S., which may limit the generalizability of the findings to other regions or populations with different environmental or dietary factors. Sixth, adjusting based on sunlight exposure was not possible as NHANES does not provide direct data on sunlight exposure. Seventh, serum vitamin D levels do not always directly reflect dietary intake, as they are influenced by multiple factors such as sun exposure, supplementation, metabolism, and genetic variations, which may confound the observed associations. However, we tried to minimize the effect of vitamin D serum level on the association between vitamin D intake and brain and mental function by adjustment in the regression model.

## Conclusion

Our study suggests that higher dietary vitamin D intake is associated with improved cognitive performance, particularly in tests related to animal fluency and memory, as well as lower levels of depression in elderly individuals. These findings indicate that adequate dietary intake of vitamin D may be linked to better cognitive function and mental well-being in older adults. However, due to the cross-sectional nature of the study, we cannot draw causal inferences. The observed associations may be influenced by unmeasured confounders or reverse causality. Future longitudinal and experimental studies are necessary to confirm these findings and explore the potential mechanisms underlying the relationship between dietary vitamin D intake and cognitive health.

## Data Availability

The datasets presented in this study can be found in online repositories. The names of the repository/repositories and accession number(s) can be found below: https://www.cdc.gov/nchs/nhanes.

## Footnotes

^1^ https://www.cdc.gov/nchs/nhanes
